# Editorial: Reviews in eating behavior: navigating complexities in health interventions, dietary practices and adolescence

**DOI:** 10.3389/fpsyg.2024.1477863

**Published:** 2024-09-17

**Authors:** Michail Mantzios, Alix Timko, Nicholas T. Bello, Daniel Rodriguez, Maria Vittoria Micioni Di Bonaventura

**Affiliations:** ^1^College of Psychology, Birmingham City University, Birmingham, United Kingdom; ^2^Department of Psychiatry, Perelman School of Medicine, University of Pennsylvania, Philadelphia, PA, United States; ^3^Department of Child and Adolescent Psychiatry and Behavioral Sciences, Children's Hospital of Philadelphia, Philadelphia, PA, United States; ^4^Department of Animal Sciences, School of Environmental and Biological Sciences, Rutgers, The State University of New Jersey, New Brunswick, NJ, United States; ^5^Department of Urban Public Health and Nutrition, School of Nursing, La Salle University, Philadelphia, PA, United States; ^6^School of Pharmacy, Pharmacology Unit, University of Camerino, Camerino, Italy

**Keywords:** eating behaviors, eating disorders, nutritional stunting, orthorexia nervosa (ON), intermittent fasting (IF)

Eating behaviors are highly influenced by the interaction of environmental and developmental factors. The purpose of this Research Topic was to highlight recent advances and possible new areas of investigation in assessing and understanding eating behaviors. The final six reviews covered a wide range of areas such as adolescent eating behaviors in countries with a high risk of nutritional stunting, risk factors for eating disorders (EDs), the impact of COVID-19 on EDs, the unintended consequences of school-based health policies, the effects of intermittent fasting over calorie restriction, and the prevalence of orthorexia in athletes.

Setiawan et al. explored literature from countries with high stunting prevalence, revealing that adolescent girls in these regions exhibit diverse and often unhealthy eating behaviors. The review found these girls engage in restrained eating, dieting for weight loss, and craving-induced eating, which contribute to low dietary diversity and micronutrient deficiencies. To address these issues, the authors make recommendations including implementing nutritional education programs, supporting positive body image, improving access to nutrient-rich foods, raising awareness about EDs, and engaging parents and communities in promoting healthy eating.

Aside from the environmental influence of malnutrition or undernutrition, Varela et al. aimed to understand adolescent eating behaviors within the broader context of EDs. The review identified significant societal and familial influences as risk factors for the onset of EDs during adolescence. Notably, the internalization of the thin ideal and living in unsupportive environments were observed as key contributors. Poor-quality relationships and being judged based on appearance were also associated with ED symptomatology. These findings emphasize the need for prevention and intervention programs that address societal pressures and foster supportive environments to improve adolescents' adaptive coping mechanisms.

Turner et al. conducted a systematic review that investigated the unintended consequences of school-based health and nutrition policies in the United States, revealing four primary themes: disordered weight control behaviors, parental discomfort or encouragement of these behaviors, ED triggers, and financial losses. The review found the impact of disordered weight control behaviors on youth to be limited and inconclusive. Parental concerns about BMI screening highlighted issues of privacy and efficacy. Although few studies addressed ED triggers, some indicated that school health classes could trigger these disorders. Additionally, one study noted increased food wastage following the replacement of sugar-sweetened beverages, which also proves problematic when considering the environmental impact of policies. These findings suggest that school health policies need a thorough evaluation to avoid adverse outcomes, with recommendations for integrating obesity and ED prevention and reconsidering BMI screenings. Overall, the reviews conducted by Setiawan et al., Varela et al., and Turner et al. collectively underline the importance of addressing unhealthy eating behaviors through comprehensive, multi-faceted approaches, involving parental and societal support, as well as targeted educational and preventive measures.

The importance of supportive environments becomes even more evident in the context of crises, such as the COVID-19 pandemic. The pandemic exacerbated EDs and maladaptive feeding behaviors, particularly among children, adolescents, and those with pre-existing conditions. McLean et al. conducted a review and found a general increase in EDs during the pandemic, likely due to the heightened stress and uncertainty that came with the presence of the pandemic. Their findings emphasize the importance of addressing mental health and illness during crises and the need for future research on understudied groups and their potential long-term implications.

Obsession with healthy eating can lead to unhealthy consequences. Paludo et al. conducted a review on orthorexia—a pathological fixation on healthy eating and food—in athletes using the ORTO-15 questionnaire. They found no significant risk of orthorexia nervosa among athletes compared to non-athletes, except for ballet dancers, who exhibited higher risk scores. Their findings suggested that orthorexia may be an issue for both athletes and general populations and highlighted the need for further evaluation beyond the ORTO-15 questionnaire. The significance of Paludo et al.'s research, in relation to previous reviews on this Research Topic, lies in its emphasis on implications for the general population, where healthy eating and nutrition need to become a sustainable lifestyle change rather than an obsession that leads to disordered eating in the name of health. Creating a holistic and balanced approach to health interventions through collaboration across different fields may lead to a solution that equally benefits both ED prevention and healthy weight regulation.

Dietary strategies, such as intermittent fasting (IF), have gained attention for their purported health benefits. In a review conducted by Gu et al., IF was found to be more beneficial than a non-intervention diet in reducing body weight, waist circumference, and adiposity while maintaining lean mass. IF also improved insulin resistance and blood lipid profiles compared to non-intervention diets. However, IF showed little benefit over caloric restriction (CR), except for reduced waist circumference. These findings suggest that while IF can be effective for weight management and metabolic health, its advantages over CR are limited. Future reviews may need to address the association between IF and CR with EDs, and, more importantly, with orthorexia. Understanding how engagement with food and the lived experiences of individuals aiming to regulate weight may enhance the understanding of creating a balanced approach to health and nutrition.

Overall, the reviews on this Research Topic present the complex and multi-faceted nature of health and nutrition interventions and eating behaviors. Addressing these issues with holistic and inclusive approaches is crucial for promoting better health outcomes across various nutritional needs and the wellbeing of “at-risk” populations.

